# Initiation of Progressive Morphological Transition Towards an Echino-Stomato-Spherocytic Phenotype by Phosphatidylserine Externalization and Its Implication in Thrombosis

**DOI:** 10.3390/ijms26041747

**Published:** 2025-02-18

**Authors:** Yiying Bian, Qiushuo Jin, Han-Young Chung, Kyung-Min Lim, Yuanyuan Xu

**Affiliations:** 1 Key Laboratory of Environmental Stress and Chronic Disease Control & Prevention Ministry of Education, School of Public Health, China Medical University, Shenyang 110122, China; 2 Key Laboratory of Liaoning Province on Toxic and Biological Effects of Arsenic, School of Public Health, China Medical University, Shenyang 110122, China; 3 Program of Environmental Toxicology, School of Public Health, China Medical University, No. 77 Pahe Road, Shenyang North New Area, Shenyang 110122, China; 4 College of Pharmacy, Seoul National University, Seoul 151-742, Republic of Korea; 5 College of Pharmacy, Chungnam National University, Daejeon 34134, Republic of Korea; 6 College of Pharmacy, Ewha Womans University, Seoul 03760, Republic of Korea; 7 Group of Chronic Disease and Environmental Genomics, School of Public Health, China Medical University, No. 77 Puhe Road, Shenyang North New Area, Shenyang 110122, China

**Keywords:** echino-stomato-spherocytic forms, phosphatidylserine exposure, thrombotic risks

## Abstract

Morphological changes in erythrocytes during disease, aging, or reactions to external agents are significant as they can influence disease progression. However, the exact mechanisms behind these temporary alterations and their potential to cause dysfunction remain unclear. Using a saponin-induced erythrocyte shape transition (EST) model, we studied the gradual shift of erythrocytes towards echino-stomato-spherocytic forms and its link to hemolysis and thrombosis. We observed that different saponin concentrations elicited varying shape transformations. At low concentrations, erythrocytes transition from discocytic shapes to echinocytic, echino-stomatocytic, and ultimately stomatocytic forms. As the concentration moderately increases, the morphology evolves into stomato-spherocytic forms. At higher saponin concentrations, the erythrocytes completely transform into spherocytic forms. Regardless of the transformation degree, all forms showed increased phosphatidylserine exposure (PS) and microvesicle (MV) production, primarily due to increased scramblase and decreased flippase activity, which were influenced by elevated calcium levels and caspase 3 activity, effectively managing PS distribution and influencing cell membrane expansion and invagination. These alterations increased thrombin production, erythrocyte adhesion, and aggregation, promoting thrombosis in rats. Altogether, our findings indicate that the shift towards echino-stomato-spherocytic forms fosters a hypercoagulable state through PS externalization, heightening thrombotic risk.

## 1. Background

With the aging of organisms, progressive morphological and functional changes will occur in various physiological processes. Among them, the morphological changes of erythrocytes are particularly noteworthy, because they not only directly participate in the transport of oxygen and nutrients, but also play a key role in maintaining the balance of blood coagulation. For instance, during the aging process or due to external stimuli, erythrocytes may undergo morphological transformation from a normal structure to various abnormal cell forms, such as echinocytes, stomatocytes, and spherocytes. The increase in these abnormally shaped erythrocytes in the blood may indicate a potential hypercoagulable state; that is, the blood coagulation tendency increases, which is prone to thrombosis. However, there is limited understanding of the relationship between morphological shifts and hypercoagulable state, which ultimately leads to an elevated risk of thrombosis.

Of note, the role of erythrocytes in thrombosis has been recently illuminated in the maintenance of hemostasis and thrombosis [1,2,3]. Erythrocytes can engage in thrombosis via procoagulant activation, facilitated by the exposure of phosphatidylserine (PS) to the outer cell membrane. Exposed PS can enable erythrocytes to promote thrombosis by providing a site for assembly of coagulation factors V and VIII to enhance the generation of thrombin, factor Xa, which is called procoagulant activity. PS-exposed erythrocytes also become more adhesive to endothelial cells, leading to vaso-occlusion, and ultimately further contributing to thrombus formation [4,5].

Hemolysis, a distinct mode of cell death in biology, serves as a reliable indicator for assessing the toxicity of external chemicals on erythrocytes. This process involves the rupturing of the cell membrane, leading to irregular and fragmented erythrocyte morphology. Normally, heme, released during erythrocyte development, functions as a DAMP, activating the immune system and triggering inflammation [6,7]. Another study, however, revealed that during hemolysis, fragmented erythrocyte debris exhibit an increased presence of externalized PS on their fragmented membranes [8]. This can further initiate thrombin generation, thereby increasing thrombotic risks. Nevertheless, the precise morphological changes remain inconsistent and require further clarification.

Under normal conditions, erythrocytes are discocytic shapes, which can be converted to echinocytic or stomatocytic shapes by a stimuli and then gradually form irreversible spherocytic shapes, leading to hemolysis [9,10]. Indeed, previous studies demonstrated that echinocytes or stomatocytes can expose PS [11,12]. During the shape changes of erythrocytes, loss of phospholipid asymmetry in the erythrocyte membrane can result in the externalization of PS and shedding of PS-exposing microvesicle (MV) [13]. Generally, the dysregulation of aminophospholipid translocases, specifically scramblase and flippase, facilitates these alterations [14]. Reactive oxygen species (ROS) [15] also play roles in PS externalization by disrupting the activities of scramblase and flippase through altering intracellular calcium level [16] caspase 3 activity [17].

Against this backdrop, we hypothesized that certain stimuli might initiate a lysis-like regulated cell death through PS externalization of intact erythrocytes, contributing to venous thrombotic risks. Here, we employed a saponin-induced erythrocytes shape transition model (EST model) to demonstrate that progressive morphological shifts towards echino-stomato-spherocytic forms occur in a concentration-dependent manner with quinine treatment. Indeed, we found that the expansion of either the inner or outer leaflet can lead to PS externalization and MV generation, resulting in increased procoagulant activity, aggravated erythrocyte aggregation, and enhanced adhesion of erythrocytes to endothelium, as well as increased formation of venous thrombi in vivo.

## 2. Results

### 2.1. Progressive Morphological Transitions in a Saponin-Induced EST Model

**Figure 1:**
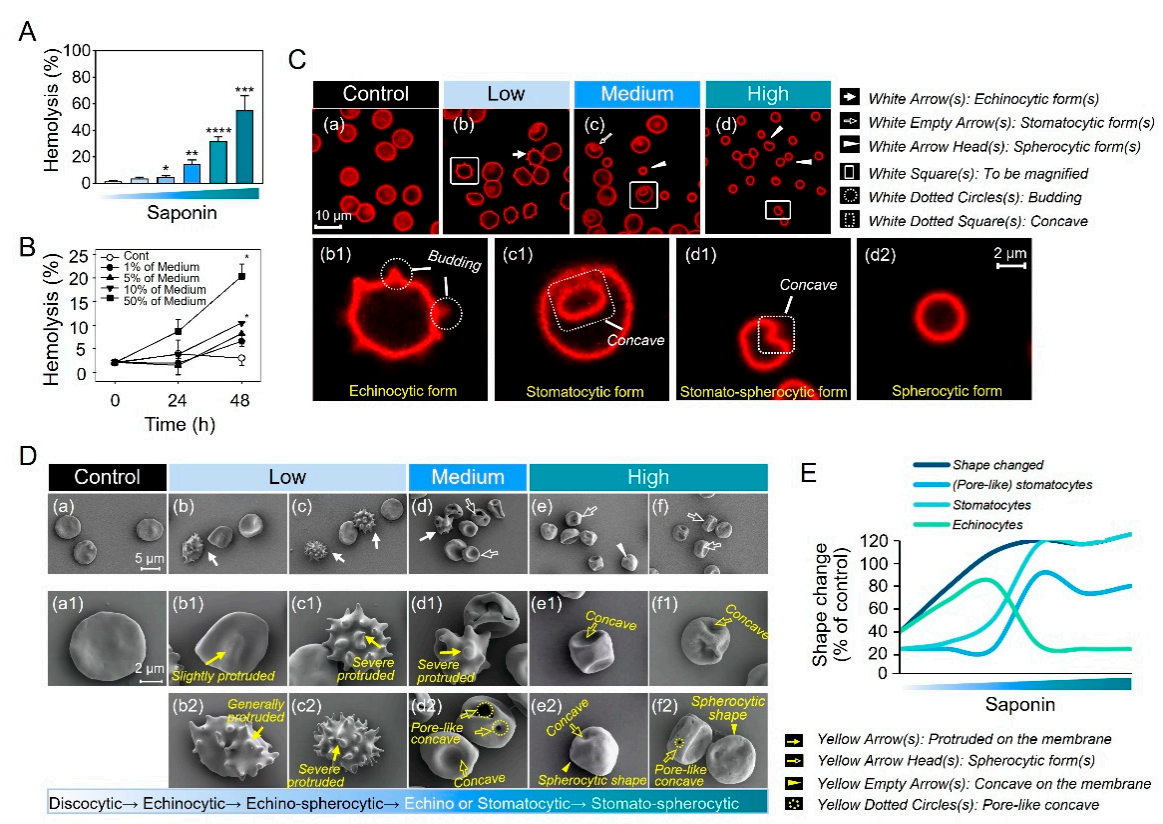
Observation of shape changes using a saponin-induced EST model. As mentioned in Methods, human erythrocytes were incubated with saponin for 24 h in various concentrations as mentioned in Methods. (**A**) Concentration-dependent hemolysis and (**B**) time-dependent (0, 24, and 48 h) hemolysis were detected at 540 nm. (**C**) Confocal microscopic observation was conducted. Erythrocyte shapes in (**C**, **a**) control, (**C**, **b**) low, (**C**, **c**) medium, and (**C**, **d**) high concentrations of saponin. (**C**, **b1**) The echinocytic form is the magnified version shown in (**C**, **b**) at a low concentration of saponin, (**C**, **c1**) the stomatocytic form is the magnified version shown in (**C**, **c**) at a medium concentration of saponin, and (**C**, **d1**) the stomato-spherocytic form and (**C**, **d2**) the spherocytic form are the magnified versions shown in (**C**, **d**) at a high concentration of saponin. White Arrow(s): Echinocytic form(s), White Empty Arrow(s): Stomatocytic form(s), White Arrow Head(s): Spherocytic form(s), White Square(s): To be magnified, White Dotted Circles(s): Budding, White Dotted Square(s): Concave. Scale bar: 2 μm. Low (**b**) means low-level treatment with 0.097 mg/mL; medium (**c**) means medium-level treatment with 0.324 mg/mL; and high (**d**) means high-level treatment with 0.97 mg/mL. Scale bar: 2 μm. (**D**) In addition, under a scanning electron microscope, shape changes were observed in control (**a**) and saponin-treated erythrocytes (**a**–**f**). (**a1**–**f2**) Each shape was magnified: (a1) the discocytic form (normal shape) shown in (**a**) control, (**b1**,**b2**) the echinocytic form with slightly and generally protruded membranes. (**c1**,**c2**) Uchino-spherocytic forms with severely protruded membranes are the magnified version shown in (**b**,**c**) low concentration treatments. (**d1**,**d2**) Both echino-spherocytic forms with severely protruded membranes and stomatocytic forms with (pore-like) concave membranes are the magnified version shown in (**d**) medium concentration treatments. (**e1**,**e2**,**f1**,**f2**) Stomatocytic forms and spherocytic shapes with (pore-like) concave membranes are the magnified version shown in (**e**,**f**) high concentration treatments. Yellow Arrow(s): Protruded on the membrane, Yellow Arrow Head(s): Spherocytic form(s), Yellow Empty Arrow(s): Concave on the membrane, Yellow Dotted Circles(s): Pore-like concave. Scale bar: 5 μm. Low (**b**,**c**) means low-level treatment with 0.097 (**b**) and 0.162 mg/mL (**c**); medium (**d**) means medium-level treatment with 0.324 mg/mL; and high (**e**,**f**) means high-level treatment with 0.97 (**e**) and 1.62 mg/mL (**f**). (**E**) The erythrocytes that underwent shape changes were counted, and their percentages relative to the control were computed. These percentages were further categorized based on their shapes and plotted accordingly. % of shape change = % echinocytes + % stomatocyts; pore-like stomatocytes is one shape of specific forms of stomatocytes. The asterisk represents significant differences from the control group (**** *p* < 0.0001; *** *p* < 0.001; ** *p* < 0.01; and * *p* < 0.05).

In the current study, we utilized various concentrations of saponin, as described in Methods, to establish a model for monitoring the gradual morphological alterations in erythrocytes under a confocal microscope. As shown in Figure 1A, shapes of erythrocytes varied by the degree of saponin treatment. Cells displayed a normal discoidal morphology before exposure to saponin. However, in response to the presence of low concentrations of saponin, some cells exhibited burr-like features characterized by budding, spicule-like structures (Figure 1C(b1), white dotted circles), which are identified as echinocytic forms (Figure 1C(b), white arrows). Interestingly, at medium concentrations of saponin, the cells exhibited a variety of distinct shapes, including a cup-like appearance (Figure 1C(c)), white empty arrows) and occasional spheroidal forms (Figure 1C(c)), white arrow heads). Upon exposure to high concentrations of saponin, nearly all the erythrocytes assumed a small, spheroidal shape (indicated by white arrowheads in Figure 1C(d)), with some of them exhibiting a concave appearance on their surface.

To gain a more detailed understanding of the morphological changes, we utilized scanning electron microscopy to observe the variations in erythrocyte morphology across different concentrations. Consistent with the confocal results, scanning electron microscopy (SEM) observations revealed the presence of echinocytes, stomatocytes, and stomato-spherocytes in the low-, medium-, and high-concentration treatments, respectively (Figure 1D(a–f)). Notably, SEM allowed for the observation of more subtle and intricate changes (Figure 1D(a1–f1) and (b2–f2)). In the low-concentration group of 0.3, cells displayed mild (Figure 1D(b1)) or moderate protrusions (Figure 1D(b2)), while in the low-concentration group of 0.5, severe protrusions were observed (Figure 1D(c1,c2)). This suggests that in the low-concentration group, the changes in echinocytes progressed gradually from mild to severe, and some of them exhibited round burr cells called echino-spherocytes (Figure 1D(c2)). In the medium-concentration group, echinocytes were rare or displayed severe protrusions with concave surface (Figure 1D(d,d1)). Most cells exhibited concave shapes on their surface, and some even displayed pore-like concave shapes (Figure 1D(d2)). In the high-concentration group (Figure 1D(e,f)), almost all cells exhibited small, rounded stomatocytic forms with significant depressions (Figure 1D(e1,e2)) or pore-like concave shapes (Figure 1D(f2)), and even appeared rough, similar to the surface of the Moon (Figure 1D(f1,f2)). Finally, the erythrocytes that underwent shape changes were counted, and their percentages relative to the control were computed. In brief, the morphological changes are highly related to the concentration of saponin treatment, and each shape across the EST model shows a progressive morphological transition towards an echino-stomato-spherocytic phenotype (Figure 1E).

### 2.2. PS Externalization Accompanies the Entire Progression of Echino-Stomato-Spherocytic Shape Changes

**Figure 2:**
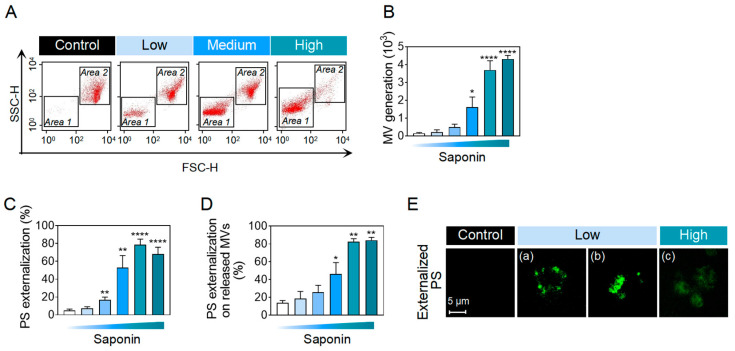
Either expansion of the inner or outer leaflet induces disrupture of the cell membrane, leading to PS exposure and MV generation. Human erythrocytes were incubated as above. (**A**) Histograms of flow cytometric analysis showed erythrocytes in Area 2 and MVs released from erythrocyte membranes in Area 1. (**B**) In Area 1, MV generation induced by saponin was increased in a concentration-dependent manner. (**C**) PS externalization on erythrocytes shown in Area 2 and (**D**) PS externalization on MVs were concentration-dependently increased. (**E**) Annexin-C-FITC was used for the observation of externalized PS on erythrocyte membranes using a confocal microscope. (**E**, **a**,**b**) show externalized PS on erythrocyte membrane in the lower group, (**E**, **c**) shows externalized PS on erythrocyte membrane in the high group, and Scale bar: 5 μm. The asterisk represents significant differences from the control group (**** *p* < 0.0001; ** *p* < 0.01; and * *p* < 0.05).

As shown in Figure 2, we employed flow cytometry to assess PS externalization on erythrocytes across various treatment grades. Figure 2A depicts two areas: Area 1 represents intact cells, while Area 2 indicates small-sized fragments known as microvesicles. As the concentration increased, microvesicles (MVs) were observed to increase in a concentration-dependent manner (Figure 2B). Furthermore, PS externalization on intact erythrocytes, which plays a crucial role in the process of thrombosis, was significantly higher in the low concentration group and increased in severity in a concentration-dependent manner (Figure 2C). Additionally, PS externalized on released MVs may also contribute to thrombosis. As saponin concentration increased, so did PS externalization on MVs (Figure 2D).

Fluorescence observations revealed that PS externalization on intact erythrocytes or MVs was evident, along with shape changes as shown in Figure 1 (Figure 2E). Interestingly, the annexin-positive signals in the low group exhibited a distinct outline resembling the cell outer membrane, suggesting PS externalization and localization on the outer leaflet of erythrocytes (Figure 2E(a)). Conversely, in the other low group, the annexin-positive signals appeared as aggregated clustered signals, possibly indicating PS externalization through cluttered PS through cell invagination (Figure 2E(b)). Lastly, in the high group, the annexin-positive signals appeared blurry and disorganized, with only a faintly visible outline of the cells (Figure 2E(c)). In brief, the PS externalization is highly related to the different morphological states (Figure S1).

### 2.3. Role of Phospholipid Translocases in Erythrocyte Morphological Alterations

**Figure 3:**
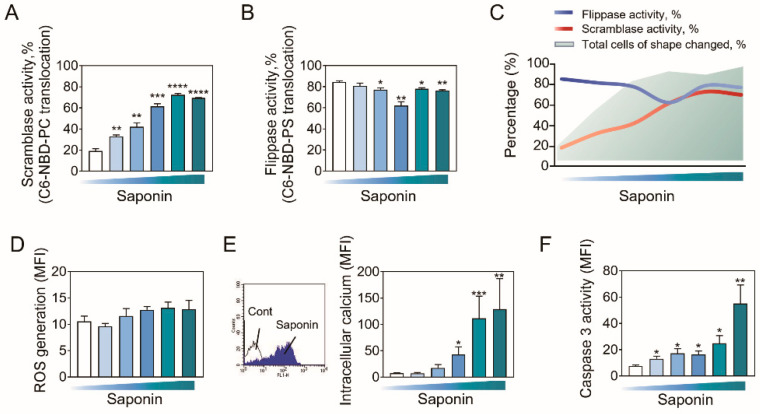
Mechanisms underlying erythrocyte morphological alterations. Human erythrocytes were incubated as above. (**A**,**B**) Scramblase and flippase activity of saponin-treated erythrocytes were evaluated by embedded C6-NBD-PC and C6-NBD-PS translocation, respectively. (**C**) A summary diagram exhibiting the relationship between percentage of total cells of shape changed (green background, data from Figure 1C) and the activity of scramblase and flippase (red line means data from Figure 3A and blue line means data from Figure 3B) in erythrocytes. (**D**) ROS generation, (**E**) intracellular calcium ([Ca^2+^]_i_) level (the histogram were inserted), and (**F**) caspase 3 activity were evaluated after 24 h incubation with various concentrations of saponin. The asterisk represents significant differences from the control group (**** *p* < 0.0001; *** *p* < 0.001; ** *p* < 0.01; and * *p* < 0.05).

Previous studies established the role of phospholipid translocases, scramblase, and flippase, in maintaining the balance of phospholipid asymmetry [9]. We examined the activities of scramblase and flippase through determining the translocations of C6-NPD-PC and C6-NPD-PS, respectively, after different concentrations of saponin treatment. As a result, scramblase activity was significantly upregulated by saponin in a concentration-dependent manner (Figure 3A), while flippase activity was gradually downregulated (Figure 3B). Figure 3C presents a general trend of the overall changes in the activities of two phospholipid translocases (blue and red line) and the total number of cells with altered morphology (green background) as saponin concentration increases.

Based upon the key role of ROS generation in PS externalization [18], we determined ROS generation after adding saponin for 24 h, but no obvious increase was observed (Figure 3D). However, Figure 3E showed a significant increase in intracellular calcium ([Ca^2+^]_i_) level, which has been reported to be one factor regulating PS externalization [19,20]. As well, caspase-3 activation has been known as another contributor to scramblase activation and initiates PS externalization [21]. Consistently, increased activity of caspase-3 was concentration-dependently observed in saponin-treated erythrocytes (Figure 3F).

### 2.4. Biological Significances of PS Externalization-Associated Shape Changes

**Figure 4:**
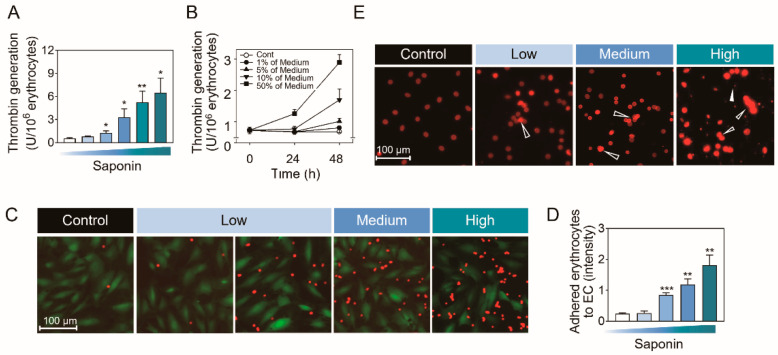
The biological significance of erythrocytes with altered shapes. Human erythrocytes were incubated as described above. (**A**,**B**) The prothrombinase assay was employed as described in the Methods section, and thrombin generation was elicited in concentration- and time-dependent trends. (**C**,**D**) At 24 h, erythrocyte-EC adhesion was concentration-dependently enhanced by saponin treatment, as observed under a fluorescence microscope. Red color: erythrocytes; green color: endothelial cells. Scale bar: 100 μm. (**E**) Erythrocyte aggregation was observed after adding glycophorin-a PE under a fluorescence microscope. Empty arrowheads: aggregated erythrocytes. Solid arrowheads: cellular debris of erythrocytes. The asterisk represents significant differences from the control group (*** *p* < 0.001; ** *p* < 0.01; and * *p* < 0.05).

Thrombin generation was significantly and concentration-dependently increased (Figure 4A). Through observing the time-dependent effect, we found that thrombin had a significant time-dependent increase, which appeared at a lower toxic dose (Figure 4B). Upon integrating the data from Figure 1A and Figure 1B, we observed that moderate hemolysis occurred in the low group, characterized by the presence of moderately blurred cells, exhibiting a moderate level of thrombin generation. Notably, severe hemolysis was evident in the medium group with concave cell shapes exhibited a high level of thrombin generation. Furthermore, erythrocyte adhesion to endothelial cells (Figure 4C,D) and erythrocyte self-aggregation (Figure 4E) were also significantly and concentration-dependently increased.

### 2.5. In Vivo Assessment Using Venous Thrombosis Rat Model

**Figure 5:**
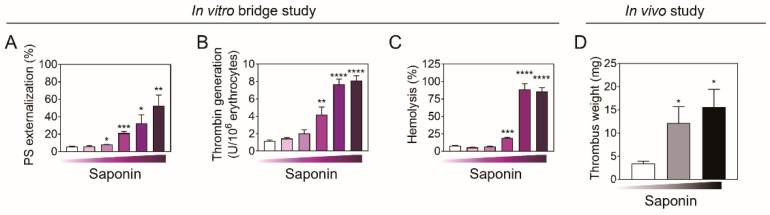
In vivo assessment of saponin in rats using a venous thrombosis model. (**A**–**C**) Freshly isolated rat erythrocytes were incubated with various concentrations of saponin for 24 h as mentioned in the Methods section. (**A**) PS externalization, (**B**) thrombin generation, and (**C**) hemolytic responses in rat erythrocytes showed the same pattern as in humans. (**D**) Increased thrombus formation was induced by saponin with i.p. administration of 0, 10, and 25 mg/kg (in saline) once daily for 4 days. Thrombus weight was measured as described in the Methods section. The asterisk represents significant differences from the control group (**** *p* < 0.0001; *** *p* < 0.001; ** *p* < 0.01; and * *p* < 0.05).

Rat erythrocytes in vitro exhibited the same pattern in the responses to quinine with those observed in human erythrocytes, as evidenced by PS externalization, thrombin generation, and hemolysis (Figure 5A–C). Most importantly, rats treated with a different dosing of saponin (i.p. with 10 and 25 mg/kg, once daily for 4 d) showed an evident increase in thrombus weight (Figure 5D), which supported in vitro data above.

## 3. Discussion

In the present study, we employed a saponin-induced EST model to investigate whether progressive morphological shifts occur concurrently with a different degree of hemolysis and an augmented procoagulant activity of erythrocytes, leading to exacerbated thrombosis. Specifically, saponin can induce concentration-dependent morphological alterations in erythrocytes, such as echinocytic, stomatocytic, and spherocytic erythrocytes, which exhibit expansion of either the inner or outer leaflet, as well as stimulate procoagulant activities through PS externalization and microvesicle generation. The activation of scramblase and inactivation of flippase play a role in the externalization of PS, which may be attributed to the elevated intracellular calcium levels and enhanced caspase 3 activation. Additionally, hemolysis increased thrombin generation in erythrocytes, erythrocyte-endothelial cell adhesion, and erythrocyte aggregation, all of which can promote thrombosis, as evidenced by the observation of increased thrombus weight in rats treated with different doses of saponin.

**Scheme 1:**
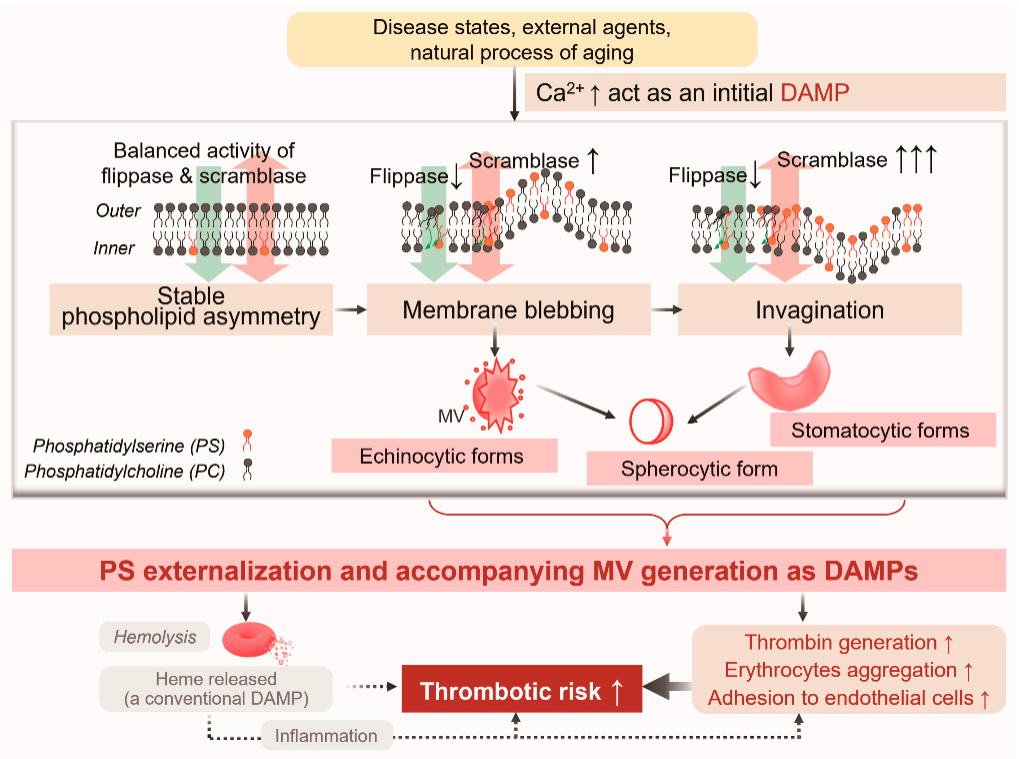
Role of PS externalization in progressive morphological changes and increased thrombotic risks. Typically, apoptosis involves the externalization of PS. In the context of the hemolysis model, the continuous exposure of PS and the subsequent release of MVs promote progressive changes in cell morphology, ultimately resulting in a lysis-like cell death. Additionally, this PS externalization and the generation of MVs function as damage-associated molecular patterns (DAMPs), further triggering the activation of coagulation cascades and ultimately elevating thrombotic risks. One upward arrow (↑) means an increase, while one downward arrow (↓) means a decrease. Three upward arrows (↑↑↑) indicate a very significant increase.

Hemolysis is widely recognized as one of the most common methods for detecting erythrocyte toxicity. It is generally believed that hemolysis can lead to the generation of irregular cell fragments and the release of heme, activating the body’s immune system and triggering an inflammatory response, ultimately leading to various diseases [6]. We also subscribed to this view in our early research on ginsenoside-induced hemolysis and irregular cell fragments in human erythrocytes, finding that such irregular fragments can also promote thrombus formation through PS externalization [8]. However, the present study is the first to propose that hemolysis-like cell death can be a regular, PS externalization-regulated, and morphologically changing process of the progressive morphological evolution of cells, and that PS and MV can further also act as damage-associated molecular patterns (DAMPs) to promote the generation of thrombin (Scheme 1).

Interestingly, erythrocyte, following the exposure to saponin, presented distinct shape changes depending on the concentrations. Previous studies have shown that shape changes from normal discocytes to echinocytes and stomatocytes are reversible, but spherocytes are irreversible and result in hemolysis [9,10]. However, it lacks the connection between morphological changes and PS externalization, as well as the lack of deep mechanism research, let alone the research on the relationship between morphological changes and diseases. In our study, during the shape alterations, we discovered that the membrane exhibiting either the expansion of the inner leaflet (stomatocytic forms) or the outer leaflet (echinocytic forms) can lead to disruptions in phospholipid asymmetry. However, despite these differences, both conditions resulted in PS externalization, ultimately triggering the procoagulant activity of erythrocytes and potentially leading to thrombosis.

Upregulation of scramblase and downregulation of flippase were attributable to the disruption of lipid asymmetry. Scramblases are known to move phospholipids nonspecifically and bidirectionally, disrupting membrane asymmetry in a Ca^2+^-dependent manner without consuming ATP. In contrast, flippases and floppases specifically transport PS and phosphatidylethanolamine (PtdEtn) to the inner membrane leaflet and phosphatidylcholine (PtdCho) in the opposite direction, respectively, using ATP energy to establish an asymmetric phospholipid distribution [22]. The regulation of the two enzymes together controls the distribution of phospholipids represented by PS, and ultimately affects the inward or outward expansion of the cell membrane, possibly leading to different morphological changes. At lower concentrations, a 2-fold increase in scramblase activity was observed with minimal effects on flippase activity, which can result in a moderate extent of PS externalization that was presented as echinocytic shapes. Indeed, during slight hemolysis, more than 3-fold increases in scramblase activity as well as significantly decreased flippase activity may ultimately lead to a considerable loss of lipid asymmetry, resulting in cell invagination and stomatocytic shapes, in which PS externalization is more extensively elicited than sub-hemolytic concentrations. Furthermore, during serious hemolysis, more than 4-fold increases in scramblase activity as well as slightly decreased flippase activity may ultimately lead to a considerable loss of lipid asymmetry, carrying out the process of PS exposed to outer membrane or cell invagination, finally reaching the step of spherocytic forms.

Additionally, during severe hemolysis, a substantial increase in scramblase activity, exceeding a 4-fold rise, along with a slight reduction in flippase activity, can potentially result in an extremely serious loss of lipid asymmetry. This process achieves a round cell state rapidly based on the completion of early PS re-distribution and cell invagination. Additionally, calcium influx and caspase activation were promoted, part of which matched well with a previous report by Mischitelli et al. [23]. Although distinct shape is involved, the perturbation of lipid symmetry occurs, which can lead to PS externalization and contribute to thrombosis.

PS externalization typically serves as a marker of apoptosis. Consequently, the molecular pathways responsible for PS exposure may encompass caspase activation, scramblase activity, IP3R-mediated calcium signaling, and sphingolipid metabolism [24,25,26]. These pathways collectively orchestrate the redistribution of PS and signal the onset of apoptotic cell death. In our study, we focus on the interplay between morphological changes and PS externalization, examining how scramblase (calcium-dependent but ATP-independent) and flippase (ATP-dependent) regulate membrane dynamics and continuously disrupt phospholipid asymmetry, ultimately leading to morphological changes of varying degrees. During this process, due to the non-specific interference of scramblase and the bidirectional regulation of phospholipid asymmetry, as well as the inherent integrity of membrane dynamics, it cannot be ruled out that other phospholipid substances, such as PtdCho, PtdEtn, phosphatidylinositol (PtdIns), etc., may concurrently undergo alterations. Additionally, the activity of flippase, another enzyme that regulates membrane asymmetry but is not specific to PS [27], might be altered in future considerations.

Clinically, echinocytes and spherocytes typically occur in long-stored erythrocytes, which may impact recovery after transfusion [28], and stomatocytes have been observed in blood films from patients with a rare disease [29]. Currently, there is very little research on the morphological changes of erythrocytes, which also limits the clinical attention given to them. Scientists made various efforts to reveal and demonstrate the importance of morphological observations; for example, previous studies have shown that spherocytic forms are irreversible and result in hemolysis [30], and a further study has shown a type of hemolysis-associated thrombotic risk [31]. In conjunction with our research, we propose that morphological alterations in erythrocytes can function as reliable, direct, and cost-effective biomarkers for hemolysis and thrombosis. Certainly, the integration of PS externalization and MV generation as rising DAMPs and future biomarkers with calcium ion measurement may enhance the reliability of thrombotic risk assessment.

Heme is a conventional DAMP, playing a pivotal role in the pathogenesis of numerous diseases. For instance, sickle cell disease is linked to endothelial activation precipitated by the release of DAMPs, including heme [32]. Heme also functions as a DAMP in platelet activation and ferroptosis, contributing to pulmonary thrombotic disease [33], and it exacerbates the thrombotic risk in β-thalassemia and sickle cell disease [34]. Thrombin generation has been employed to elucidate coagulation mechanisms that remain incompletely understood and to investigate hyper- or hypocoagulability in clinical scenarios associated with an elevated risk of thrombosis or hemorrhage [35]. Consistently, PS externalization and MV generation mechanically contribute to thrombin generation [36]. Our study suggests that PS externalization and MV generation can, to some extent, act as DAMPs, promoting thrombin generation and contributing to thrombotic risk. Furthermore, we propose that employing PS-blocking strategies could potentially mitigate the progression and incidence of such thrombotic risks based on the previous successful attempts in various disease [37,38,39]. Under normal physiological conditions, erythrocytes typically measure approximately 7.5 microns in diameter and 2 microns in thickness. However, in various pathological states, the morphology of erythrocytes can be significantly altered, with a loss of size and thickness. For example, in cases of anemia, iron deficiency anemia, thalassemia, and other related diseases, erythrocytes may exhibit abnormal morphologies. These aberrant forms include echinocytes, spherocytes, elliptocytes, stomatocytes, sickle cells, schistocytes, and acanthocytes, among others [40]. The EST model used in this study, which focused on observing echinocytes, stomatocytes, and spherocytes, did not include observations or studies of other morphological changes. This could be due to the transient nature of other morphologies, making them challenging to capture, or it may indicate that the saponin-induced EST model is not effective in inducing these other morphological changes. This finding suggests that morphological alterations induced by different exogenous chemicals may vary and possess a certain degree of specificity. Therefore, more different models are needed to study the relationship between morphology and disease and molecular mechanism. Nonetheless, whether similar mechanisms, such as PS exposure, could elevate the risk of thrombosis remains unclear and warrants further, more rigorous investigation. Consequently, in a clinical setting, if common morphological abnormalities are detected, it is imperative to pay attention to these findings and issue appropriate risk warnings.

In summary, we demonstrated that a progressive morphological shifts from normal discocytic forms to echinocytic, stomatocytic, and spherocytic forms were shown in different degree of hemolysis, and all of these performed different degrees of PS externalization and MV generation, which can further induce procoagulant activity of erythrocytes, ultimately leading to thrombosis. Our study serves to bridge the research gap by offering a deeper understanding of temporary morphological changes observed in clinical or experimental settings. Specifically, it addresses the limitations of previous studies by exploring the underlying mechanisms and potential functional abnormalities that may underlie these changes and ultimately lead to diseases. By doing so, our work contributes to a more comprehensive and nuanced understanding of these complex biological phenomena.

## 4. Materials and Methods

### 4.1. Materials

Parent dapsone, CaCl_2_, glucose, ethylenediaminetetraacetic acid (EDTA), bovine serum albumin (BSA), N-[2Hydroxyethyl]piperazine-N’-[2-ethanesulfonic acid] (HEPES), sodium dodecyl sulfate, Triton X-100, and purified human thrombin were the chemicals provided by Sigma-Aldrich Co., based in St. Louis, Missouri, USA. BD Pharmingen (San Diego, CA, USA) provided the fluorescein isothiocyanate (FITC)-labeled annexin V (an-nexin V-FITC) and the phycoerythrin-labeled monoclonal mouse anti-human CD235a (an-ti-glycophorin-A-PE). 1-Palmitoyl-2-[6-[(7-nitro-2-1,3-benzoxadiazol-4-yl)amino], hexa-noyl]-snglycero-3-phospho-L-serine (C6-NBD-PS), and 1-oleoyl-2-[6-[(7-nitro-2-1,3-benzoxadiazol-4-yl)amino]hexanoyl]-sn-glycero-3-phosphocholine (C6-NBD-PC) were from Avanti Polar Lipids (Alabaster, AL, USA). Hematologic Technologies, Inc. (Essex Junction, VT, USA) provided the purified human prothrombin (factor II), while Chromogenix (Milano, Italy) supplied S2238, and Lonza (Basel, Switzerland) supplied the endothelial cell growth media (EGM) kit and human umbilical vein endothelial cells (HUVECs). Calcein-green AM was from Invitrogen (Carlsbad, CA, USA). Quinine hydrochloride dihydrate was purchased from Sigma-Aldrich Co., based in St. Louis, MO, USA.

### 4.2. Erythrocytes Preparation

With the approval from the Ethics Committee of Health Service Center at Seoul National University (IRB No.1702/003-004), human blood was obtained from healthy male donors (20–30 years old) using a vacutainer with acid citrate dextrose and a 21 gauge needle (Becton Dickinson, Franklin Lakes, New Jersey, USA) on the day of each experiment. Centrifugation at 200 g for 15 min was followed by aspiration to remove platelet-rich plasma and buffy coat. Phosphate buffered saline (PBS: 1.06 mM KH_2_PO_4_, 154 mM NaCl, and 2.96 mM Na_2_HPO_4_ at pH 7.4) and Ringer’s solution (125 mM NaCl, 5 mM KCl, 1 mM MgSO_4_, 32 mM HEPES, 5 mM glucose, and pH 7.4) were used to wash the packed erythrocytes twice. Prior to use, the final CaCl_2_ concentration was adjusted to 1 mM after washed erythrocytes were resuspended in Ringer’s solution to a cell concentration of 5 × 10^7^ cells/mL. Here, we created an erythrocyte shape transition model (EST model) induced by saponins using varying quinine concentrations. After being treated with various concentrations of saponin for 24 h at 37 °C (control with DW, low-level treatment with 0.097 and 0.162 mg/mL, medium-level treatment with 0.324 mg/mL, and high-level treatment with 0.97 and 1.62 mg/mL), the erythrocytes were examined.

### 4.3. Detection of Hemolytic Activity

Spectrophotometric measurements at 540 nm were used to determine the degree of hemolysis after samples were centrifuged (10,000× *g* for 1 min) following erythrocyte incubation with chemicals. Triton X-100-lyzed erythrocytes were utilized for 100% hemolysis.

### 4.4. Confocal Microscopic Observation

An erythrocyte suspension (3 × 10^6^ cells/mL) was added to a four-chambered coverslip (Lab-Tek^®^ from Thermo Fisher, NY, USA) and allowed to attach completely for one hour at room temperature. After washing with Ringer’s solution containing 2% BSA to remove any detached cells, chemicals suspended in the solution were added. Ringer’s solution containing anti-glycophorin A-PE was used to stain erythrocytes for 30 min following incubation. Confocal microscopy with an argon laser (TCS SP8, Leica, Germany) was used to observe any morphological changes. The emission and excitation filters were positioned at 550–600 nm and 488 nm, respectively.

### 4.5. Flow Cytometric Analysis

The markers for PS detection and erythrocyte identification were annexin V-FITC and anti-glycophorin A-PE, respectively. Negative controls for annexin V binding were stained with annexin V-FITC in the presence of 2.5 mM EDTA instead of 2.5 mM CaCl_2_. The FACS Calibur flow cytometer (Becton Dickinson, USA) with an argon-ion laser that emits at 488 nm was used to analyze the samples. A log scale was used to set the light scatters and fluorescence channels. Software called Cell Quest Pro version 5.1 was used to gather and analyze data from 5000 events. Forward scatter characteristics were used to identify PS following calibration with 1% standard beads. Analysis was possible for both PS externalization in the MV and erythrocyte areas.

After phospholipid translocation was detected, chemically activated erythrocytes were exposed to 0.5 μM C6-NBD-PC (for scramblase activity) and C6-NBD-PS (for flippase activity) for 0, 15, 30, and 60 min at 37 °C. The fluorescence intensity associated with the cells before (without 1% bovine serum albumin) and after (with 1% bovine serum albumin) back extraction on ice for 10 min was compared in order to calculate the amount of internalized probe. Data from 5000 events were gathered and examined with Becton Dickinson’s Cell Quest Pro software.

### 4.6. SEM Observation

The erythrocytes were fixed with a 2% glutaraldehyde solution for one hour at 4 °C, centrifuged, and then washed three times with PBS before being post-fixed with 1% osmium tetroxide for 30 min at room temperature in the hood. Following two PBS washes, the samples were serially dehydrated using 50%, 70%, 80%, 90%, and 100% ethanol. These images were examined using a field emission scanning electron microscope (Merlin Compact FE-SEM, Zeiss, Oberkochen, Germany) after being dried and coated with gold.

### 4.7. Prothrombinase Assay

After treatment with chemicals, samples were incubated with 5 nM factor Xa and 10 nM factor Va in Tyrode buffer (134 mM NaCl, 10 mM HEPES, 5 mM glucose, 2.9 mM KCl, 1 mM MgCl_2_, 12 mM NaHCO_3_, 0.34 mM Na_2_HPO_4_, 0.3% BSA, and 2 mM CaCl_2_ at pH 7.4) for 3 min at 37 °C. Exactly 3 min after the addition of 2 μM prothrombin, an aliquot of the suspension was transferred to a tube containing stop buffer (50 mM Tris-HCl, 120 mM NaCl, and 2 mM EDTA at pH 7.9). The chromogenic substrate S2238 (chromogenic substrate for thrombin; Chromogenix, Milano, Italy) was used to measure thrombin activity. An active-site-titrated thrombin calibration curve was used to determine the rate of thrombin formation from the change in absorbance at 405 nm.

### 4.8. Fluorescence Microscopy Observation

Erythrocyte-HUVECs adhesion experiments were conducted as follows: Endothelial cells (2 × 10^4^ cells) were seeded into a 4-well-chamber for 2 days and stained with calcein green for 20 min. Chemical-exposed erythrocytes (resuspended in EBM-2 to 5 × 10^7^ cells/mL) were layered onto confluent calcein green-stained HUVEC monolayer for 1 h at 37 °C. Glycophorin A-PE was used to stain the remaining erythrocytes after nonadherent erythrocytes were removed using EBM-2. The adhesion of erythrocytes to HUVECs was then observed under fluorescent microscopy. Additionally, fluorescence microscopy was used to observe the aggregation of chemically exposed erythrocytes following the addition of glycophorin A-PE.

### 4.9. In Vivo Experiments

All the protocols were approved by the Institutional Animal Care and Use Committee of the Animal Service Center at Seoul National University (IACUC No. SNU-170417-27-4). Sprague Dawley rats (300–400 g, purchased from SamTako Co., Osan, Korea) were anesthetized with urethane (1.25 g/kg, i.p.), blood (Acid-citrate-dextrose as anticoagulants) was collected from abdominal aorta, and erythrocytes were isolated as human method for in vitro rat study. Then, hemolysis, PS externalization, and thrombin generation were examined as mentioned above. For the experiment of thrombus formation, we referred to the method in the previous study [11].

### 4.10. Statistical Analysis

The means and standard errors of means were calculated for all treatment groups (more than four independent experiments). The data were subjected to one-way analysis of variance followed by Duncan’s multiple range test to determine which means were significantly different from the control. In all cases, a *p* value of < 0.05 was used to determine significant differences.

## Data Availability

The data presented in this study are available upon request from the corresponding author.

## Supplementary Materials

The following supporting information can be downloaded at: https://www.mdpi.com/article/10.3390/ijms26041747/s1.
