# Supplementary figures and images for: Initiation of Progressive Morphological Transition Towards an Echino-Stomato-Spherocytic Phenotype by Phosphatidylserine Externalization and Its Implication in Thrombosis
Source: Int J Mol Sci. 2025 Feb 18;26(4):1747. doi: 10.3390/ijms26041747 (PMC11854928; doi:10.3390/ijms26041747)

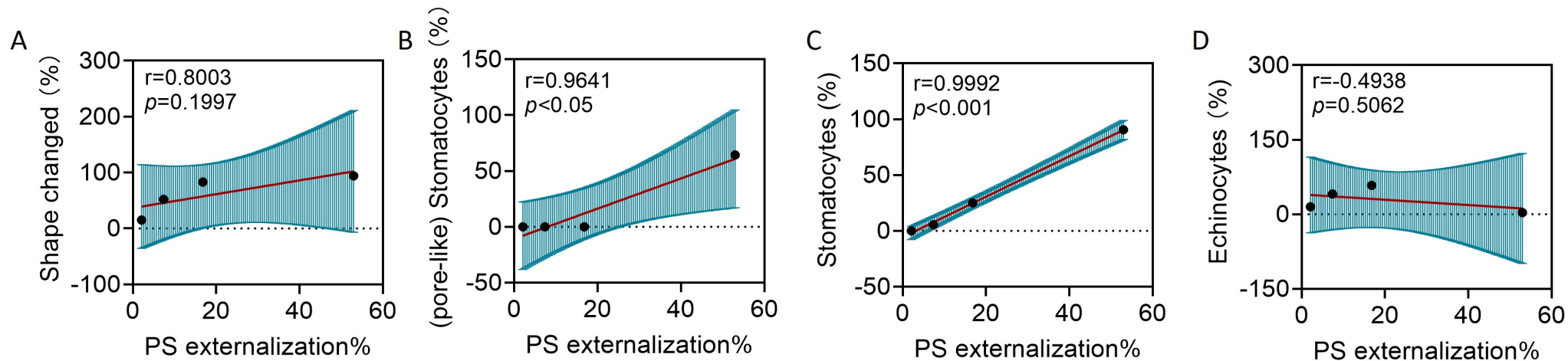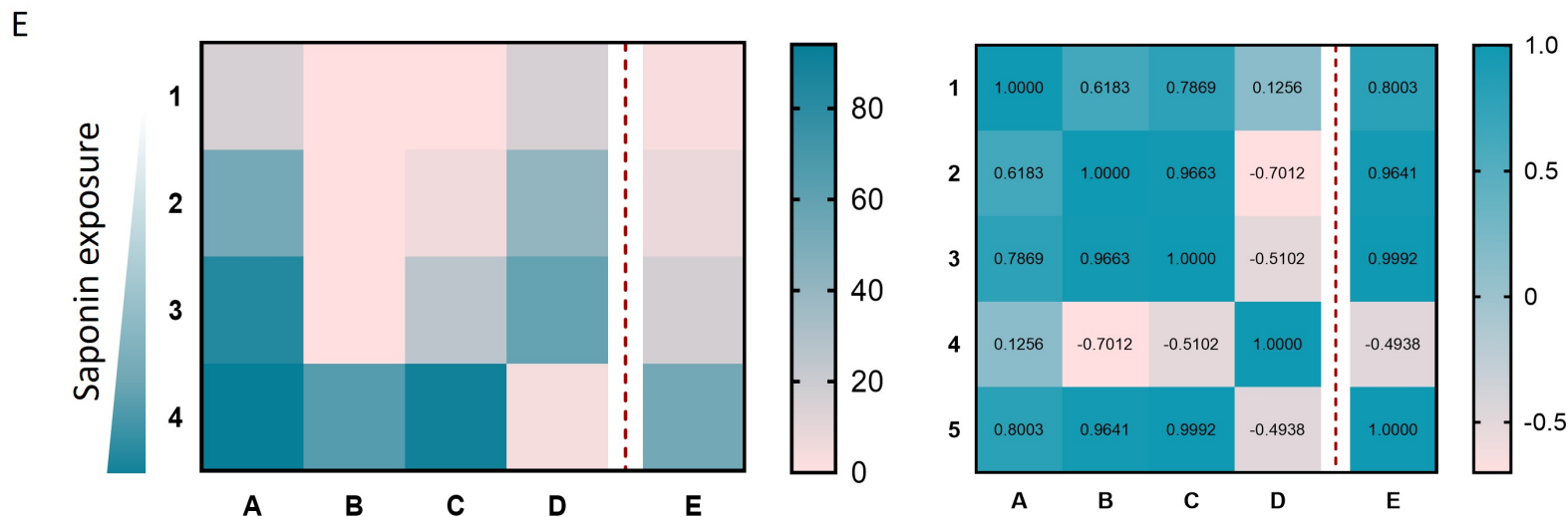

A: Shape changed% B: (pore-like) Stomatocytes%; C: Stomatocytes%; D: Echinocytes%; E:PS externalization%

Supplement: Supplementary file 1 [file ijms-26-01747-s001.zip › ijms-3423711-supplementary.pdf]
